# Detection of p53 aggregates in plasma of glioma patients

**DOI:** 10.1038/s43856-025-00918-3

**Published:** 2025-05-23

**Authors:** Yunzhao Wu, Jeff Y. L. Lam, Matthaios Pitoulias, Dorothea Böken, Ziwei Zhang, Renuka Chintapalli, Emre Fertan, Zengjie Xia, John S. H. Danial, Gemma Tsang-Pells, Emily Fysh, Linda Julian, Kevin M. Brindle, Richard Mair, David Klenerman

**Affiliations:** 1 Yusuf Hamied Department of Chemistry, University of Cambridge, Cambridge, UK; 2 UK Dementia Research Institute, University of Cambridge, Cambridge, UK; 3 Department of Clinical Neurosciences, University of Cambridge, Cambridge, UK; 4 Cancer Research UK Cambridge Institute, University of Cambridge, Cambridge, UK

**Keywords:** CNS cancer, Diagnostic markers

## Abstract

**Background:**

The tumour-suppressor protein p53 can form amyloid aggregates resulting in loss of tumour-suppressing functions and leading to tumour formation. The detection of p53 aggregates in cancer cells has been demonstrated but these aggregates have not been detected in liquid biopsies to date, due to the lack of sufficiently sensitive methods.

**Methods:**

We developed an ultrasensitive immunoassay based on the single-molecule array (SiMoA) technology to detect p53 aggregates in plasma, based on antibody capture of the aggregates. We confirmed that the assay detects p53 aggregates using super-resolution imaging. We then investigated the p53 aggregate concentrations in the plasma of 190 pre-surgery glioblastoma (GB) patients and 22 controls using this assay.

**Results:**

We found that the plasma p53 aggregate levels are significantly elevated in pre-surgery GB patients’ plasma compared to controls. Longitudinal study further reveals that p53 aggregate levels may increase before GB recurrence and decrease following treatment. We also observed raised p53 aggregate concentrations in the plasma of cancer patients with brain metastases.

**Conclusions:**

This study demonstrates the detection of p53 aggregates in liquid biopsies. Our findings highlight the potential of p53 aggregates as a novel biomarker for glioblastoma.

## Abstract (web summary)

Wu et al. develop an ultrasensitive immunoassay based on the single-molecule array (SiMoA) technology to detect p53 aggregates in plasma. They show that the p53 aggregate concentrations in the plasma of pre-surgery glioblastoma patients are significantly higher compared to controls.

## Plain language summary

p53 is an important protein molecule that prevents cancer from developing. However, under some conditions, p53 can clump together to form aggregates. These aggregates cannot stop cancer and may even help cancer grow. This makes p53 aggregates a potential marker of cancer. We have developed a very sensitive test to catch and detect the rare p53 aggregates in the blood. With this test, we find that there are more p53 aggregates in the blood of glioblastoma (a highly aggressive brain cancer) patients than people without cancer. Our test is 90.57% accurate in diagnosing glioblastoma and may also help predict cancer recurrence and monitor treatment response.

## Introduction

p53 is a tumour suppressor protein that protects genome integrity by regulating a wide range of cellular processes, such as DNA repair^1^, cell cycle arrest^2^, and apoptosis^3^. Given its broad tumour-suppressing functions, *TP53* is the most frequently mutated gene in human cancer, and the production of mutant p53 protein is intimately associated with oncogenesis^4^. Wild-type (WT) and some mutant forms of p53, such as the R248Q and R175H mutants, can form amyloid aggregates that lose the tumour-suppressing functions of p53 and exert tumour-promoting activities^5–8^. p53 aggregation has been documented in various cancers, including neuroblastoma^6^, breast cancer^7^, and ovarian cancer^8^. Understanding the implications of p53 aggregation can provide new insights into cancer pathology and therapy.

Glioblastoma (GB) is one of the most aggressive and recurrent brain tumours, with patients having an average survival of 14.6 months and a 5-year survival of 7.2%^9^. Although there is currently no cure for GB, detection of GB in its nascent, low-grade form may improve patient survival, underscoring the urgent need for an effective diagnostic biomarker^10^. Overall, the *TP53* gene is mutated in ~28% of GB patients, and the mutation rate of *TP53* in different GB subtypes can vary from 0% to 54%^11,12^, suggesting that p53 may play a critical role in GB development. Notably, the ARF-p53-MDM2 pathway, an important tumour suppressor pathway, is dysregulated in 84% of GB patients and 94% of GB cell lines^11^. Overexpression of mutated p53, including the aggregation-prone mutants, is common in GB and may lead to the accumulation of p53, and, subsequently, the formation of p53 aggregates^13^. Moreover, p53 aggregates have been found to promote chemoresistance in GB cells, suggesting a potential relationship between p53 aggregation and GB malignancy^14^. Therefore, p53 aggregates may serve as a promising diagnostic and prognostic biomarker for GB.

The observation of p53 aggregates in GB cells and in other cancer cell lines^14–16^ suggests that p53 aggregates may be detectable in plasma collected by venesection (liquid biopsy). Here, we developed an ultrasensitive assay targeting p53 aggregates in human plasma based on single-molecule array (SiMoA) technology, an immunoassay capable of detecting proteins at femtomolar concentrations (10^−15^ M)^17–21^. Using the SiMoA platform, we aimed to explore the utility of p53 aggregates in plasma as a diagnostic biomarker of GB, as well as an indicator of GB recurrence and therapy response.

## Methods

### Plasma and tissue sample collection and processing

Patients were recruited at Addenbrooke’s Hospital, Cambridge, UK as part of the Integrated Clinically-Augmented Repository for Universal Sampling (ICARUS) tissue collection which is an ethically-approved Research Tissue Bank (REC 18/EE/0172). All sample collection involved in this study was approved by the institutional ethics committee. Patients with suspected GB on pre-operative contrast-enhanced MRI were chosen for participation in the study. Sampling was performed during the initial surgery for a new diagnosis of glioma. Plasma from control patients undergoing non-cancer surgery was collected intra-operatively (matching conditions for the GB cohort). The control patients were matched age and sex to the GB cohort. The diagnoses of the controls are shown in Supplementary Table S1. Follow up samples were collected during outpatient clinics with MRI being performed as per routine clinical paradigm. Stable disease and tumour recurrence were defined by contrast enhanced MRI performed every three months as per clinical regimen. Recurrence was defined as per RANO criteria^22^. We used a pragmatic approach to identify the largest cohort possible for these analyses. Informed consent was obtained from all subjects, and all experiments conform to the principles set out in the Declaration of Helsinki. Samples were centrifuged at 1500 × *g* at 4 °C for 10 min before being aliquoted into 0.5-mL protein lo-bind microcentrifuge tubes (Eppendorf, Cat. No. 0030108094). The aliquoted samples were stored at −80 °C. All plasma samples underwent a maximum of three freeze-thaw cycles before being tested.

To determine the total protein concentration of the plasma samples, all samples were first diluted 10-fold in phosphate-buffered saline (PBS, Gibco, Cat. No. 10010023). The total protein concentrations were then determined by A_280_ (NanoDrop One, Thermo Scientific, Cat. No. ND-ONE-W).

### SiMoA bead conjugation

The antibody-conjugated SiMoA bead was prepared following the manufacturer’s instructions. Firstly, 100 μg of DO-1 antibody (Abcam, Cat. No. Ab1101) was buffer-exchanged into the Bead Conjugation Buffer (Quanterix, Cat. No. 101354) using an AmiconUltra-0.5 centrifugal filter (Merck, Cat. No. UFC500396, molecular weight cut-off 50 kDa). The buffer-exchanged antibody was then diluted with the Bead Conjugation Buffer to 0.2 mg/mL and kept on ice before use. Secondly, a total of 4.2 × 10^8^ carboxylated paramagnetic beads (Quanterix, Cat. No. 101354) were transferred to a 1.5-mL microcentrifuge tube (Eppendorf, Cat. No. 0030108116). The tube was then placed on a magnetic separator (Cytiva, Cat. No. 28-9489-64) for 1 min, and the supernatant was removed. The beads were washed three times with the Bead Wash Buffer (Quanterix, Cat. No. 101354) and another three times with the Bead Conjugation Buffer. Subsequently, the washed beads were activated using a freshly prepared solution of 0.3 mg/mL 1-ethyl-3-(3-dimethylaminopropyl)carbodiimide (EDC, Thermo Scientific, Cat. No. A35391) in the Bead Conjugation Buffer at 4 °C for 30 min on a HulaMixer (Thermo Scientific, Cat. No. 15920D). Thirdly, the activated beads were washed once with the Bead Conjugation Buffer, followed by the addition of the buffer-exchanged antibody. The reaction mixture was incubated at 4 °C for 2 h on the HulaMixer. Next, the antibody-conjugated beads were washed twice with the Bead Wash Buffer and blocked with the Bead Blocking Buffer (Quanterix, Cat. No. 101354) for 45 min at room temperature on the HulaMixer. Finally, the blocked beads were washed twice with the Bead Wash Buffer, resuspended with 300 μL of Bead Diluent (Quanterix, Cat. No. 101354), and stored at 4 °C until use.

### Preparation of recombinant p53 aggregate

The recombinant p53 protein was purchased from GenScript (WT p53: 1.10 mg/mL, GenScript, Cat. No. U0276EA140-5/P4EB001; R248Q p53: 1.41 mg/mL, GenScript, Cat. No. U0276EA140-9/P4EB001). The recombinant p53 aggregates were prepared by diluting the stock proteins using the aggregation buffer (50 mM Tris, 150 mM NaCl, 5 mM 1,4-dithiothreitol (DTT, Roche, Cat. No. 10708984001), pH 7.2) to 1 μM. The protein samples were then incubated at 37 °C on a shaking incubator (Grant-bio) for 72 h, sonicated in an ice-water bath for 5 min, and diluted with the aggregation buffer to the indicated concentrations.

### Preparation of brain homogenate

GB tumour tissue was obtained after surgery. Tissue homogenisation was performed using a previously established protocol. Briefly, the homogenisation buffer was freshly prepared by dissolving one tablet of phosphatase inhibitor (Merck, Cat. No. 4906845001, 1 tablet/10 mL) and one tablet of protease inhibitor (Merck, Cat. No. 5892791001, 1 tablet/10 mL) into a solution containing 10 mM Tris-HCl, 0.8 M NaCl, 1 mM EGTA, 0.1% Sarkosyl, and 10% sucrose (pH 7.32). The brain homogenisation buffer was then filtered by a 0.22 μm syringe filter. To each homogenisation tube (Merck, Cat. No. Z763721-50EA), 120 mg of frozen tissue was added, followed by the addition of 1.2 mL of ice-cold homogenisation buffer. The homogenisation tube was then placed on a VelociRuptor V2 Microtube Homogeniser (Scientific Laboratory Supplies, Cat. No. SLS1401), which was then operated at 5 m/s for two cycles of 20 s each, with a 10-s rest between the cycles at 4 °C. Subsequently, the tube was centrifuged at 4 °C at 21,000 × *g* for 20 min and the supernatant was collected in a clean microcentrifuge tube (Merck, Cat. No. EP0030108450-1EA). Next, 0.6 mL of ice-cold homogenisation buffer was added to the homogenisation tube, followed by VelociRuptor shaking using the same programme and centrifugation at 21,000 × *g* for 20 min at 4 °C. The supernatant was combined with the previously collected supernatant fraction. The total protein concentrations of the brain homogenate samples were measured by BCA assay following the manufacturer’s instructions (ThermoFisher, Cat. No. 23225).

### SiMoA assay

The SiMoA assay was performed following the manufacturer’s instructions. Briefly, to each well of a 96-well plate (Quanterix, Cat. No. 103077), 20 μL of plasma and 80 μL of Homebrew Sample/Detector diluent (Quanterix, Cat. No. 101359) were loaded, followed by the addition of 25 μL of DO-1-conjugated beads in Bead Diluent (2 × 10^7^ beads/mL). The maximum sample volume in the preliminary experiments was set to 25 μL per well. For assay optimisation, the sample consisted of either 20 μL of plasma mixed with 80 μL of diluent, or 5 μL of plasma and 95 μL of diluent. Plasma samples were sonicated in an ice-water bath for 5 min before being introduced to the plate. For brain homogenate samples, 2 μL sample + 98 μL Sample/Detector diluent or 5 μL sample + 95 μL Sample/Detector diluent was added to each well. The samples and calibrators were tested in duplicates. The 96-well plate was then incubated on a shaking incubator for 30 min at 30 °C, 800 rpm. Subsequently, the plate was washed using a microplate washer (Quanterix). Meanwhile, the biotinylated PAb240 (Novus Biologicals, Cat. No. NB200-103B) detector antibody was diluted with the Sample/Detector diluent to 0.3 μg/mL. After the first wash, 100 μL of the detector antibody was introduced to each well. The plate was then incubated for 10 min on the shaking incubator before returned to the washer for the second wash. The SBG (Quanterix, Cat. No. 101361) was diluted with the SBG diluent (Quanterix, Cat. No. 101361) to 150 pM. Next, 100 μL of the SBG solution was introduced to each well and the plate was incubated for 10 min on the shaking incubator. Finally, the plate was washed again and loaded on the SiMoA SR-X detection system for analysis.

### Preparation of the calibrator

The 15-nm 3-aminopropyl(3-oxobutanoic acid) functionalised silica nanoparticles (SiNaP-COOH, Merck, Cat. No. 660450, estimated concentration 11.47 µM) were centrifuged at 10,000 × *g* at room temperature for 1 h. The supernatant was discarded, and the pellet resuspended in an equal volume of 2-(*N*-morpholino)ethanesulfonic acid (MES) buffer (10 mM, pH 5.7). Meanwhile, 1-ethyl-3-(3-dimethylaminopropyl) carbodiimide (EDC, ThermoFisher, Cat. No. A35391) and sulfo-*N*-hydroxysuccinimide (sulfo-NHS, ThermoFisher, Cat. No. A39269) were freshly dissolved in cold MES buffer (10 mM, pH 5.7) at 10 and 20 mg/mL, respectively. These solutions were mixed with the SiNaP-COOH suspension to achieve final concentrations of 100 nM SiNaP-COOH, 400 μM EDC, and 100 μM sulfo-NHS. The mixture was sonicated for 30 min, centrifuged at 10,000 × *g* for 1 h at room temperature, and the pellet resuspended in 1 mL of 10 mM MES buffer at 200 nM. The DO-1-binding peptide (sequence: K(PEG)_8_TFSDLWKLLP, Cat. No. U577SIB280-5) and the PAb240-binding peptide (sequence: K(PEG)_8_TFRHSVV, Cat. No. U577SIB280-7) were separately dissolved in water to give 1 mM concentrations. To 1 mL of the activated SiNaP-COOH suspension, a mixture of 5 µL DO-1-binding peptide solution and 5 µL PAb240-binding peptide solution was added. The reaction mixture was placed on a HulaMixer and incubated overnight at room temperature and centrifuged at 10,000 × *g* for 1 h at 4 °C. The pellet was resuspended in 1 mL of 1:1 H_2_O:DMSO (v/v), sonicated for 5 min, and centrifuged at 5,000 × *g* for 1 h at 4 °C. Finally, the pellet was resuspended in 200 µL of 1:1 H_2_O:DMSO (v/v), aliquoted, and stored at −20 °C until use. The stock concentration of the peptide-conjugated SiNaP was estimated to be 1 µM, assuming no loss during the reactions. The synthesis route is shown in Supplementary Fig. S1.

### Prediction of aggregation propensity

The aggregation propensities of the DO-1-binding peptide (sequence: TFSDLWKLLP), PAb240-binding peptide (sequence: TFRHSVV), and their concatenated peptide (sequence: TFSDLWKLLPTFRHSVV), were input into the PASTA2.0 website and analysed with default thresholds for peptides. The top pairing energy and the energy threshold were set to 1 and −5, respectively.

### Data analysis

The calibration curve was fitted using the four-parameter logistic (4PL) fit with 1/*y*^2^ weighting. The p53 aggregate concentrations in the samples were back-calculated from the mean AEB values of two duplicate wells using the calibration curve. The lower limit of detection was back-calculated from the mean ± 2.5 standard deviation (SD) of the blank AEB. The normalised p53 aggregate concentrations were calculated by dividing the fitted concentration (pM) by the protein A_280_ of the same sample.

### Statistics and reproducibility

All analyses were performed using the OriginLab 2021b software (OriginLab Corporation, USA). Normally distributed data were compared using Student’s *t* test. Mann–Whitney *U*-test was performed for datasets that are not normally distributed. *P* values below 0.05 are considered statistically significant.

For SiMoA experiments, all plasma samples were tested in two individual wells on the same plate and the readout of one sample was the mean of the two wells. The total protein concentration (as in A_280_) of each sample was the mean of two measurements. For diffraction-limited imaging, each data point represents the mean of nine field-of-views (FOVs) within the same well. For super-resolution imaging, each data point represents the mean of two FOVs within the same well. The patient samples were imaged once. The recombinant p53 aggregates were prepared and imaged three times independently.

### SiMPull of p53 aggregates in plasma

The SiMPull coverslips were prepared as previously described^23,24^. The coverslips were firstly cleaned by three 10-min sonication periods in 18.2 MΩ·cm water, acetone (Thermo Fisher, Cat. No. 10442631), and methanol (Thermo Fisher, Cat. No. 10675112). The coverslips were then sonicated in 1 M KOH solution for 20 min, thoroughly rinsed with methanol, water, and methanol, dried with a stream of nitrogen, and cleaned with argon plasma for 15 min (PDC-002, Harrick Plasma). Subsequently, the coverslips were silanised with a 3:5:100 mixture of 3-aminopropyl triethoxysilane (Fisher Scientific UK, Cat. No. 10677502), acetic acid (Merck, Cat. No. 45726), and methanol, and sonicated in two cycles, each comprising a 60 s ‘ON’ period and a 10 min ‘OFF’ period. The silanised coverslips were again rinsed with excess methanol, water, and methanol before being dried with a stream of nitrogen. Next, a 50-well PDMS gasket (Merck, Cat. No. GBL103250) was carefully affixed to each coverslip. Meanwhile, a 100:1 aqueous mixture of methoxy-PEG-Succinimidyl Valerate (110 mg/mL, Laysan Bio Inc., Cat. No. MPEG-SVA-5000) and biotin-PEG-Succinimidyl Valerate (100 mg/mL, Laysan Bio Inc., Cat. No. Biotin-PEG-SVA-5000) was freshly prepared. Each well on the coverslip was passivated with 9 µL of the mixture and 1 µL of 1 M NaHCO_3_ solution (pH 8.5). The coverslips were then incubated in a humidity chamber overnight at room temperature, rinsed with excess 18.2 MΩ·cm water, and dried with a stream of nitrogen. Each well was further passivated by introducing a mixture of 9 µL of 10 mg/mL methyl-PEG4-NHS-Ester (Thermo Fisher, Cat. No. 22341) and 1 µL of 1 M NaHCO_3_ solution (pH 8.5). The coverslips were again incubated overnight in the humidity chamber, washed with 18.2 MΩ·cm water, and dried with a stream of nitrogen. Finally, the dried coverslips were stored in a desiccator at −20 °C until use.

The SiMPull assay was performed as previously described^23^. Prior to the experiment, the following buffers and reagents were prepared and stored on ice: (1) PBST: PBS supplemented with 0.05% tween 20 (Merck, Cat. No. P1379-100ML); (2) PBS + 1% tween: PBS supplemented with 1% tween 20; (3) PBS-BSA: PBS supplemented with 0.1 mg/mL bovine serum albumin (BSA, Thermo Fisher, Cat. No. B14). The PBST and PBS + 1% tween buffers were filtered with a 0.02 μm filter (VWR, Anotop 25, Cat. No. 516-1501). In addition, the capture antibody (DO-1, Abcam, Cat. No. Ab1101) was biotinylated with sulfo-NHS-LC-LC-Biotin (Thermo Scientific, Cat. No. 21338) following the manufacturer’s instructions. The biotinylated capture antibody and fluorescently labelled imaging antibody (Alexa Fluor 647-conjugated PAb240, Novus Biologicals, Cat. No. BS200-103AF647) were diluted with PBS-BSA to 10 and 1 nM, respectively, and stored on ice before use.

To each well of the SiMPull coverslip, 10 μL of 0.2 mg/mL neutravidin (Thermo Fisher, Cat. No. 31000) in PBST was added and incubated for 10 min at room temperature. The wells were then washed twice with 10 μL of PBST and once with 10 μL of PBS + 1% tween (three-step washing). Next, 10 μL of 10 nM biotinylated capture antibody in PBS-BSA was added, incubated for 10 min at room temperature, and washed using the same three-step washing method. The wells were then blocked with 1 mg/mL BSA in PBS for 15 min at room temperature and washed. Samples (10 μL/well) were then added and incubated overnight at 4 °C, followed by three-step washing. Plasma samples were sonicated for 5 min in an ice-water bath and diluted 1:1 in PBS. The recombinant p53 aggregates were prepared using the same protocol as in the SiMoA assay and diluted with the aggregation buffer to 1 nM. The monomeric WT and R248Q p53 samples were prepared by mixing the 1 μM protein solutions 1:3 with 8 M guanidine hydrochloride solution (pH 8.5, Merck, Cat. No. G7294-100ML). The mixture was then incubated overnight at 4 °C and further diluted using the aggregation buffer to a final protein concentration of 1 nM. After the three-step washing, the wells were again blocked with 1 mg/mL BSA in PBS for 15 min and washed. Then, the 1 nM imaging antibody in PBS-BSA was introduced to all wells, incubated for 10 min, and removed by three-step washing. Finally, the dSTORM imaging buffer (50 mM cysteamine, 10% glucose, 50 mM Tris, 10 mM NaCl, 0.5 mg/mL glucose oxidase, 40 μg/mL catalase, pH 8.0) was freshly prepared and introduced to all wells^25^. The wells were sealed with a clean coverslip and imaged immediately. All incubation steps were performed in a humidity chamber.

### Fluorescence imaging

Fluorescence imaging was performed on a home-built total internal reflection fluorescence (TIRF) microscope, using a flat-field illumination module with four lasers (405 nm: Oxxius, LBX-LD; 488 nm: Cobolt, MLD 488; 561 nm: Roithner LaserTechnik, RLTMLL-561-100-3; 638 nm: Cobolt, MLD 638)^26^. The laser beams were cleaned by the corresponding excitation filters (405 nm: FF01-417/60-25, Semrock; 488 nm: LL01-488-25, Semrock; 532 nm: FF01-532/3-25, Semrock; 638 nm: FF01-640/14-25, Semrock) and coupled into a 70-μm square-core optical fibre (05806-1 Rev. A, CeramOptec) using an aspheric lens (C220TMD-A, Thorlabs). The optical fibre was agitated using a vibrational motor (304-111, Precision Microdrives) operating at 1.5 V for speckle removal. The laser output from the fibre was collimated with an adjustable collimator (C40FC-A, Thorlabs), cleaned by a quadband excitation filter (FF01-390/482/563/640-25×36, Semrock), focused by an achromatic lens (AC254-125-A-ML, Thorlabs), and reflected by a pentaband dichroic beam splitter (R405/488/561/635/800-T1-25×36, Semrock) into an oil-immersion objective (×100 CFI Apo TIRF, NA 1.49, MRD01991, Nikon) mounted on a microscope body (Ti-E Eclipse, Nikon). The fluorescence from the sample was collected by the objective and cleaned by a quadband emission filter (FF01-446/523/600/677-25×36, Semrock) and a short-pass filter (FESH0750, Thorlabs). The fluorescence was further cleaned by corresponding emission filters (405 nm: FF01-480/40-25, Semrock; 488 nm: FF03-525/50-25, Semrock; 561 nm: FF01-600/37-25, Semrock; 638 nm: LP02-647RU-25, Semrock) before being recorded on a sCMOS camera (Prime BSI Express, Teledyne Photometrics). The pixel size was 87.21 nm under two-by-two binning. All devices were controlled using MicroManager (μManager v1.4.22)^27^. Automated imaging was achieved using a home-written script.

dSTORM imaging was performed under the illumination of 405 nm laser (43 mW) and 638 nm laser (110 mW), the power densities of which were approximately 430 and 1100 W/cm^2^, respectively. For each well of a SiMPull coverslip, two FOVs were imaged. For plasma samples, the imaging was performed once. For p53 monomer and aggregate samples, three replicates were used. A total of 5000 frames were obtained for each FOV with 50 ms exposure time. The images were analysed using the ThunderSTORM plugin in ImageJ/FIJI^28,29^. Localisations with intensities below 200 and uncertainties above 20 nm were filtered out. The cluster analysis was performed using density-based spatial clustering of applications with noise (DBSCAN) with the *ε* and *min_sample* set to 75 nm and 5, respectively^30^. All localisation data were normalised to the blank (0.1 mg/mL BSA) on the same coverslip.

### Bulk RNA transcriptomic comparison

The tumour samples were obtained after surgery and prepared for sequencing from fresh tissue (whole genome sequencing/whole transcriptome sequencing, WGS/WTS). A matched blood sample from the same patient was used for DNA Tumour-Normal sequencing comparison. The Illumina DRAGEN v4 pipeline was used. RNA quantification was performed with Dragen/salmon pseudoalignment re-implementation and aggregated at gene level. The aggregated gene counts were normalised for library depth and RNA composition using DESeq2 and then the effect sizes (log fold changes) were shrunk with apeglm. GB Samples with a purity of less than 0.2 and a ploidy of <1.5 and >2.5 were excluded as to compare similar ploidy status. The Wald test from DESeq2 was used for statistical analysis. *TP53* mutation encapsulates *TP53* alterations spanning from single nucleotide variants (SNVs) to structural variations (SVs), fusions, copy number alterations, and loss of heterozygosity (LOH) events. Data analysis was performed in R.

### Reporting summary

Further information on research design is available in the Nature Portfolio Reporting Summary linked to this article.

## Results

### Assay development and optimisation

**Fig. 1:**
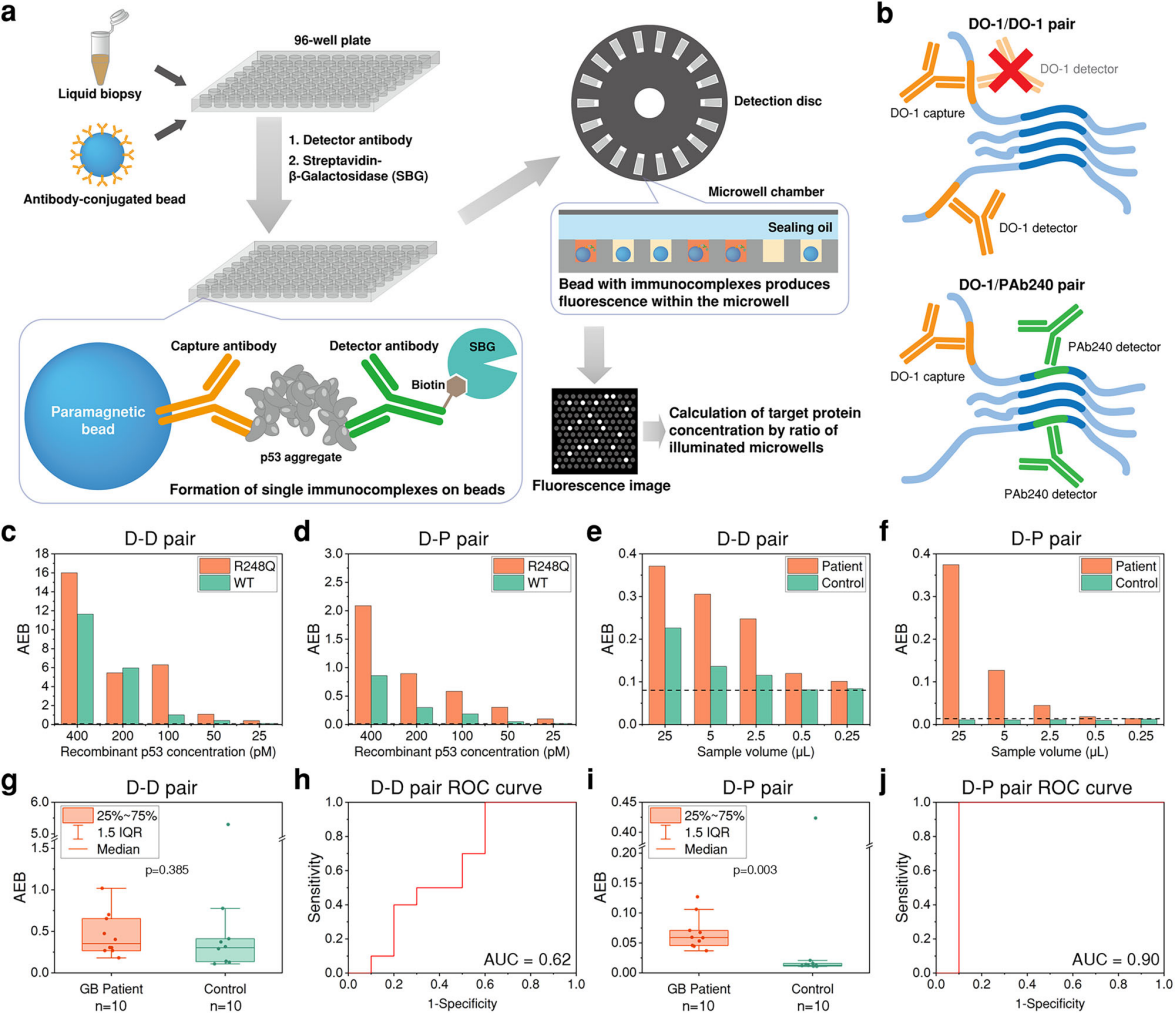
Development of the SiMoA assay. **a** Schematic illustration of the SiMoA assay. **b** Antibody pair selection. The DO-1/DO-1 (D-D) pair (top) can only detect non-monomeric p53 because the same epitope cannot bind to two identical antibodies simultaneously. The DO-1/PAb240 (D-P) pair (bottom) can detect p53 aggregates because the PAb240 antibody targets an epitope that only exposes in unfolded p53 (aa 211–217), which is present in p53 aggregates. **c**, **d** Average enzyme per bead (AEB) values of recombinant WT and R248Q p53 aggregates with the D-D and D-P pairs. The blank levels are indicated by the dotted lines. **e**, **f** AEB values of the GB and control plasma samples with the D-D and D-P pairs. The blank levels are indicated by the dotted lines. **g** AEB values of 10 GB patients and 10 controls using the D-D pair. **h** ROC curve of the D-D pair. **i** AEB values of 10 GB patients and 10 controls using the D-P pair. **j** ROC curve of the D-P pair.

The SiMoA assay relies on a pair of monoclonal antibodies to capture and detect p53 aggregates in plasma samples (Fig. 1a). Specifically, SiMoA utilises antibody-conjugated paramagnetic beads to capture target proteins in the sample, followed by incubation with a biotinylated detector antibody and streptavidin-β-galactosidase fusion protein (SBG), forming single immunocomplexes on the bead. The beads are then introduced to a microwell array, such that each microwell contains only one or no bead. The immunocomplex-carrying bead in the microwell can catalyse the hydrolysis of a fluorogenic substrate, resorufin β-D-galactopyranoside (RGP), enabling the quantification of p53 aggregate concentration in the sample. Here, we employed two p53 antibodies, DO-1 and PAb240. DO-1 recognises an epitope close to the N-terminal (aa 20–25) of p53 that is present in both native and mutant conformations^31^. In contrast, PAb240 binds to an epitope (aa 211–217) in the central DNA-binding region of p53 that is only accessible in the mutant conformation of p53^31^. We initially evaluated the performance of two capture-detector pairs, DO-1 capture/DO-1 detector (D-D) and DO-1 capture/PAb240 detector (D-P) (Fig. 1b). The D-D pair cannot detect p53 monomers due to identical epitope binding. In contrast, the D-P pair lacks the selectivity of the D-D pair for multimers but can detect p53 aggregates as aggregation requires partial unfolding of the protein, exposing the PAb240-binding epitope^32^.

We first compared the performance of the D-D and D-P pairs using recombinant p53 aggregates (Fig. 1c, d) and plasma samples from a GB patient and a control (Fig. 1e, f). The readout value of the SiMoA platform is the average enzyme per bead (AEB), which is derived from the ratio of the number of fluorescent ‘on’ beads to the total number of beads in the assay^33^. It should be noted that, in this study, the SiMoA assay works in a digital fashion where the AEB values were dependent on the number of p53 aggregates but not affected by the aggregate size distribution. In other words, the AEB values were derived from binarised microwell readouts (i.e., a microwell is considered ‘on’ if its intensity is above a certain threshold, regardless of the true intensity value). Samples containing higher concentrations of the target protein will have more beads with immunocomplexes containing enzymes and hence more microwells will be fluorescent, leading to a higher AEB. The AEB value can be converted to a concentration using a calibration sample. The AEB values were higher for recombinant aggregates with the D-D pair than the D-P pair (Fig. 1c, d). Both pairs showed increased signal levels from the R248Q p53 aggregates compared to the WT p53 aggregates, since the R248Q p53 mutant has a higher aggregation propensity compared to WT p53^34^. The patient sample showed comparable signal with both pairs, whereas the control sample showed signal levels significantly higher than the background with the D-D pair but not the D-P pair (Fig. 1e, f). Reducing the sample volume lowered the AEB values for both pairs, with the control signal for the D-P pair retained at a background level. The D-P pair showed much better discrimination of the patient samples than the D-D pair.

The performance of both pairs was further compared using 10 control and 10 GB patient samples (Fig. 1g–j). The D-D pair showed no significant difference between the controls and GB patient samples (Fig. 1g) (Control vs GB (Median (IQR)): 0.303 (0.134, 0.502) vs 0.354 (0.268, 0.665), Mann–Whitney *U*-test *p* value = 0.385), whereas the D-P pair showed a significant difference (Fig. 1i) (Control vs GB (Median (IQR)): 0.013 (0.011, 0.017) vs 0.059 (0.045, 0.080), Mann–Whitney *U*-test *p* value = 0.003). A single control sample exhibited anomalously high AEB values with both D-D and D-P pairs. The receiver operating characteristic (ROC) curve demonstrated an area under the curve (AUC) of 0.62 and 0.90 for the D-D and D-P pairs, respectively (Fig. 1h, j), suggesting that the D-P pair has a superior diagnostic accuracy to the D-D pair for p53 aggregate detection in liquid biopsies.

### Validating the presence of p53 aggregates using super-resolution imaging

To further characterise the p53 aggregates detected by the SiMoA assay, we utilised super-resolution imaging on plasma samples using a single-molecule pull-down (SiMPull) assay. SiMPull captures the proteins of interest on an antibody-coated glass coverslip for microscopic characterisation^35,36^. Similar to the SiMoA assay, we immobilised DO-1 antibodies on PEGylated coverslips to selectively ‘pull down’ p53 proteins in the plasma samples. The captured p53 aggregates were then labelled with fluorophore-conjugated PAb240 antibody for fluorescence imaging. We imaged a total of 8 GB samples with high p53 concentrations as detected by SiMoA and 9 control samples showing no p53 aggregates.

**Fig. 2:**
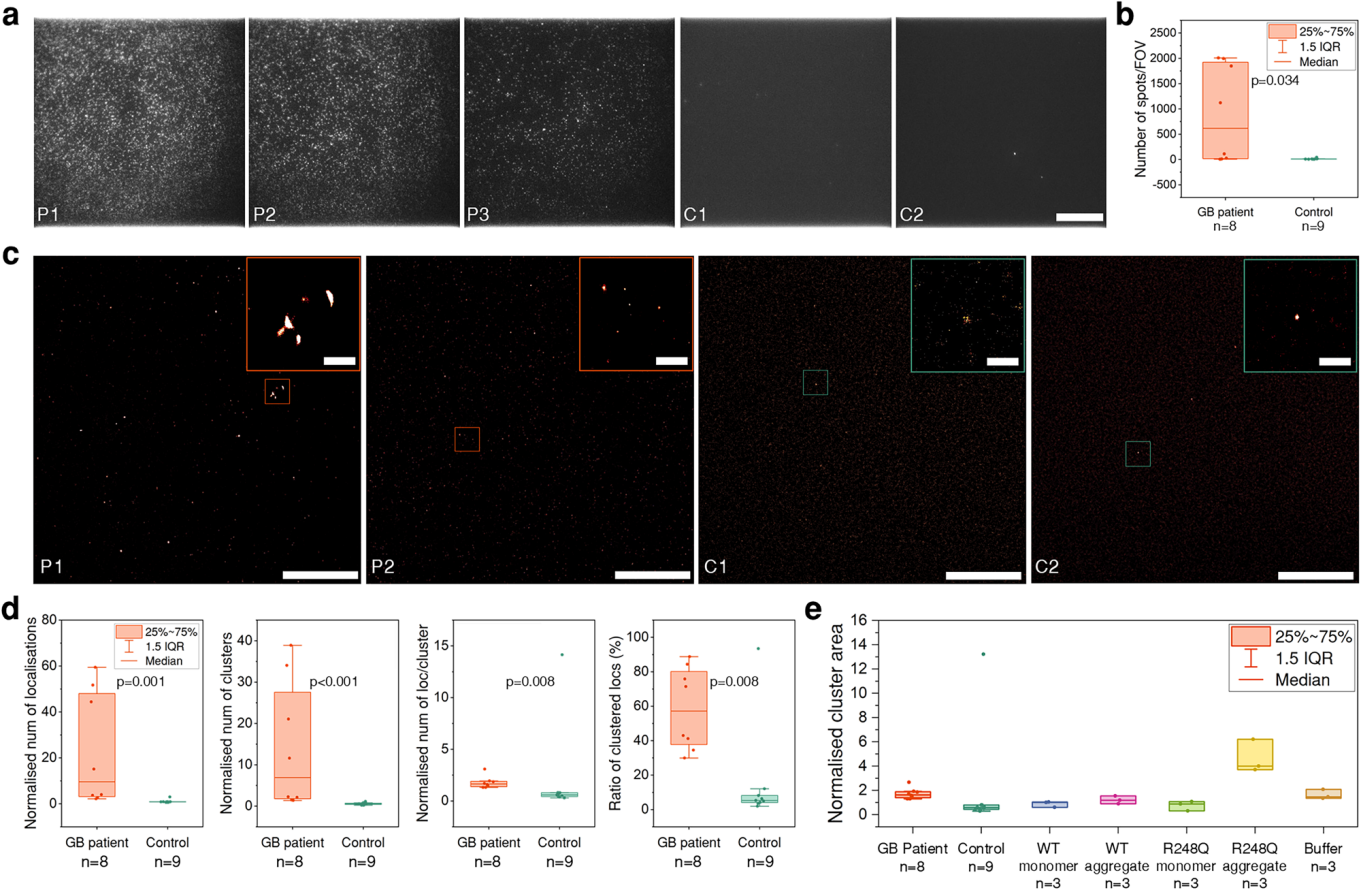
Validation of the presence of p53 aggregates in GB patient plasma. **a** Diffraction-limited images of the plasma samples of three GB patients (P1, P2, P3) and two controls (C1, C2) on the SiMPull surface. The p53 aggregates in the sample were captured using the DO-1 antibody and then labelled with the fluorescent PAb240 antibody (i.e., the D-P pair). All images share the same scale bar. The scale bar represents 20 μm. **b** Number of fluorescent spots per FOV in the diffraction-limited images. **c** Super-resolution images of the plasma samples of two GB patients (P1, P2) and two controls (C1, C2). The scale bars in the full images and the insets represent 10 and 1 μm, respectively. **d** Comparison between the total number of localisations, number of clusters, number of localisations per cluster, and ratio of localisations in clusters of 8 GB patients vs 9 controls. The data were normalised to the corresponding values of the blank wells on the same coverslip. **e** Comparison between the mean cluster area between the plasma samples and recombinant p53 monomers and aggregates. Each data point of the plasma sample represents one individual, whereas each data point of the recombinant protein sample represents one experimental replicate. The data were normalised to the corresponding values of the blank wells on the same coverslip.

Numerous fluorescent spots can be observed in the diffraction-limited images of patient plasma samples but not in the control samples (Fig. 2a), in agreement with the SiMoA results. Significantly more fluorescent spots per field-of-view (FOV) were observed in the GB plasma samples than the controls (Control vs GB (Median (IQR)): 9.75 (7.38, 14.00) vs 617.25 (15.00, 1960.13), Mann–Whitney *U*-test *p* value = 0.034) (Fig. 2b). Super-resolution imaging further demonstrated the presence of aggregates over 200 nm across in the patient samples (Fig. 2c). Cluster analysis of the localisation data showed significantly higher total area of clusters in the GB plasma samples than the controls (Control vs GB (Median (IQR)): 0.246 (0.178, 0.547) vs 9.557 (2.927, 53.402), Mann–Whitney *U*-test *p* value = 0.002) (Supplementary Fig. S2). The number of localisations per FOV (Control vs GB (Median (IQR)): 0.864 (0.716, 0.864) vs 9.576 (2.923, 49.859), Mann–Whitney *U*-test *p* value = 0.001), number of clusters per FOV (Control vs GB (Median (IQR)): 0.561 (0.316, 0.751) vs 6.925 (1.638, 30.780), Mann–Whitney *U*-test *p* value = 6.355 × 10^−4^), average number of localisations per cluster (Control vs GB (Median (IQR)): 0.592 (0.405, 0.808) vs 1.638 (1.353, 1.918), Mann–Whitney *U*-test *p* value = 0.008), and ratio of clustered localisations (Control vs GB (Median (IQR)): 5.269 (3.813, 10.119) vs 57.210 (36.153, 82.255), Mann–Whitney *U*-test *p* value = 0.008) were also higher in the GB plasma samples compared with controls (Fig. 2d). When comparing the mean cluster areas of plasma samples with those of recombinant WT and R248Q p53 monomer and aggregates at 1 nM, we found that most control samples had similar cluster areas to those of monomeric WT or R248Q p53; while the GB patient samples exhibited higher cluster areas comparable to those of the 1 nM WT p53 aggregates (Fig. 2e). Notably, one control sample showed exceptionally high mean cluster areas and localisations per cluster, while the buffer showed higher mean cluster areas than WT p53 monomers and aggregates. This was because the control and buffer samples had very small number of clusters compared with patient or recombinant protein samples, and a few relatively large clusters in the images of these samples could result in abnormally high average cluster areas or number of localisations per cluster. These large, but very rare, clusters may be induced by debris, surface defect, or non-specific binding of the detector antibody. These data collectively suggest that more and larger p53 aggregates exist in the GB patients’ plasma, which may be responsible for the elevated signals in the SiMoA assays.

### Calibrator development and assay optimisation

**Fig. 3:**
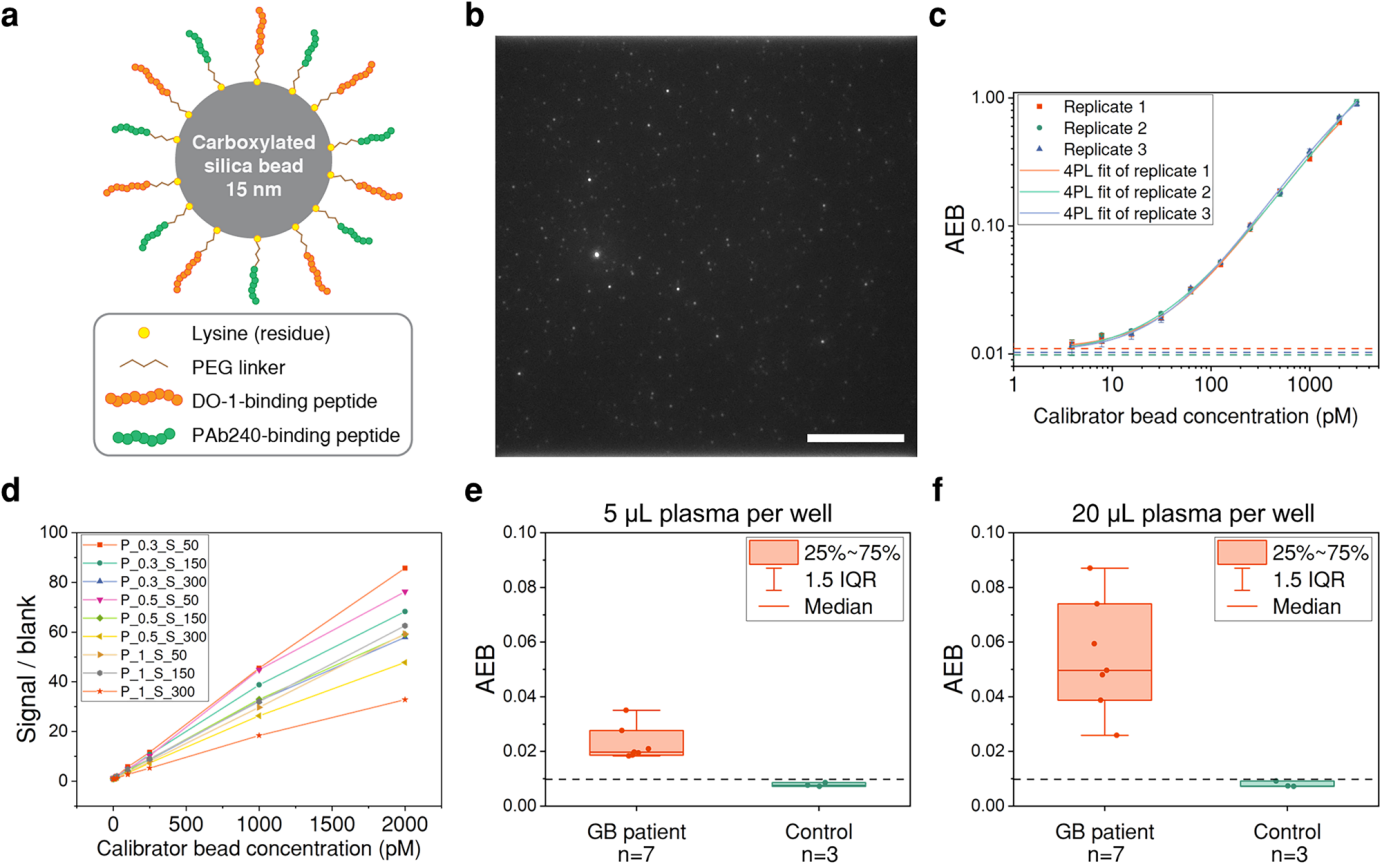
Calibrator development and assay optimisation. **a** Illustration of the calibrator. The DO-1- and PAb240-binding peptides are conjugated to a silica nanoparticle to mimic a p53 aggregate. An amide bond is formed between the lysine residue on the peptide and the carboxyl group on the silica nanoparticle. **b** Microscopic image of the calibrator labelled with fluorescent PAb240-antibody. The scale bar represents 20 μm. **c** Calibration curves of three experimental replicates. The blank levels are indicated by the dotted lines of corresponding colours. The error bars denote the standard deviations of three on-plate replicates. **d** Signal-to-background ratios (SBR) of different detector-SBG concentration combinations. ‘P’ and ‘S’ stand for PAb240 and SBG, respectively, and their concentrations are indicated with the following numbers. For example, ‘P_0.3_S_50’ stands for 0.3 μg/mL PAb240 + 50 nM SBG. **e**, **f** AEB values obtained from 5 and 20 μL sample volumes. The blank levels are indicated by the dotted lines.

We next developed a calibrator for the SiMoA assay based on the D-P antibody pair, which enables calculation of p53 aggregate concentrations from the AEB values and can compensate for experimental variation and ensure consistency in the results. Since recombinant p53 is prone to aggregation and can form aggregates that are heterogeneous in size and structure^34^, it was not suitable as a calibration standard. We therefore developed a calibrator composed of silica beads conjugated with the epitopes targeted by the DO-1 and PAb240 antibodies. The two peptides are covalently attached to the silica nanoparticle and are thus unlikely to inter- or intra-molecularly interact with each other to form aggregates. Hence, this single entity peptide-coated silica nanoparticle can mimic a fixed size aggregate with multiple antibody-binding peptides (Fig. 3a)^34^. We first evaluated the aggregation propensity of the two peptides using the online amyloid prediction platform, PASTA2.0^37^. Both peptides and their concatenated sequence were predicted not to form amyloid aggregates (amyloids = 0), suggesting a low likelihood of either self-aggregation or cross-aggregation. Fluorescence images of the calibrator beads also showed no significant aggregation (Fig. 3b). In SiMoA experiments, the calibrator bead showed highly consistent AEB values in three experimental replicates (Fig. 3c). The calibrator exhibited a large linear range spanning from approximately 4 to 3000 pM, covering AEB values between 0.01 and 1, within which the SiMoA operates in the ‘digital’ mode for aggregate detection^33^.

To improve the sensitivity and diagnostic accuracy of the assay, we further optimised the SiMoA assay conditions, including detector concentration, SBG enzyme concentration, and sample volume. We chose the detector and SBG concentrations based on SiMoA assay development instructions provided by Quanterix, which suggested using 0.3 μg/mL and 150 pM for initial assay development. Therefore, we used 0.3, 0.5, and 1.0 μg/mL detector concentrations to investigate whether higher concentrations could enhance the assay sensitivity; we used 50, 150 and 300 pM for SBG to investigate which concentration is optimal in terms of signal-to-background ratio (SBR, calculated by AEB_Sample_/AEB_Blank_). The SBRs for various detector-SBG concentration combinations are shown in Fig. 3d. The lower detection limit and AEB range for all conditions were calculated and are shown in Supplementary Table S2. Most conditions had similar background levels, suggesting that the lower limit of detection of the assay may be limited by non-specific binding. Among the three conditions with the highest SBR (0.3 μg/mL detector + 50 pM SBG; 0.5 μg/mL detector + 50 pM SBG; and 0.3 μg/mL detector + 150 pM SBG), 0.3 μg/mL detector + 150 pM SBG had the largest AEB value at maximum calibrator bead concentration, implying a large linear region. A sample volume of 20 μL per well differentiated control and GB patient samples better than using 5 μL (Fig. 3e, f). While a higher sample volume per well may lead to larger differences between controls and patients, we chose 20 μL per well due to the limited total volume of sample available. Therefore, 0.3 μg/mL capture detector, 150 pM SBG, and 20 μL sample per well were considered to be optimal for detection of p53 aggregates.

### Detection of p53 aggregates in GB patient plasma

**Fig. 4:**
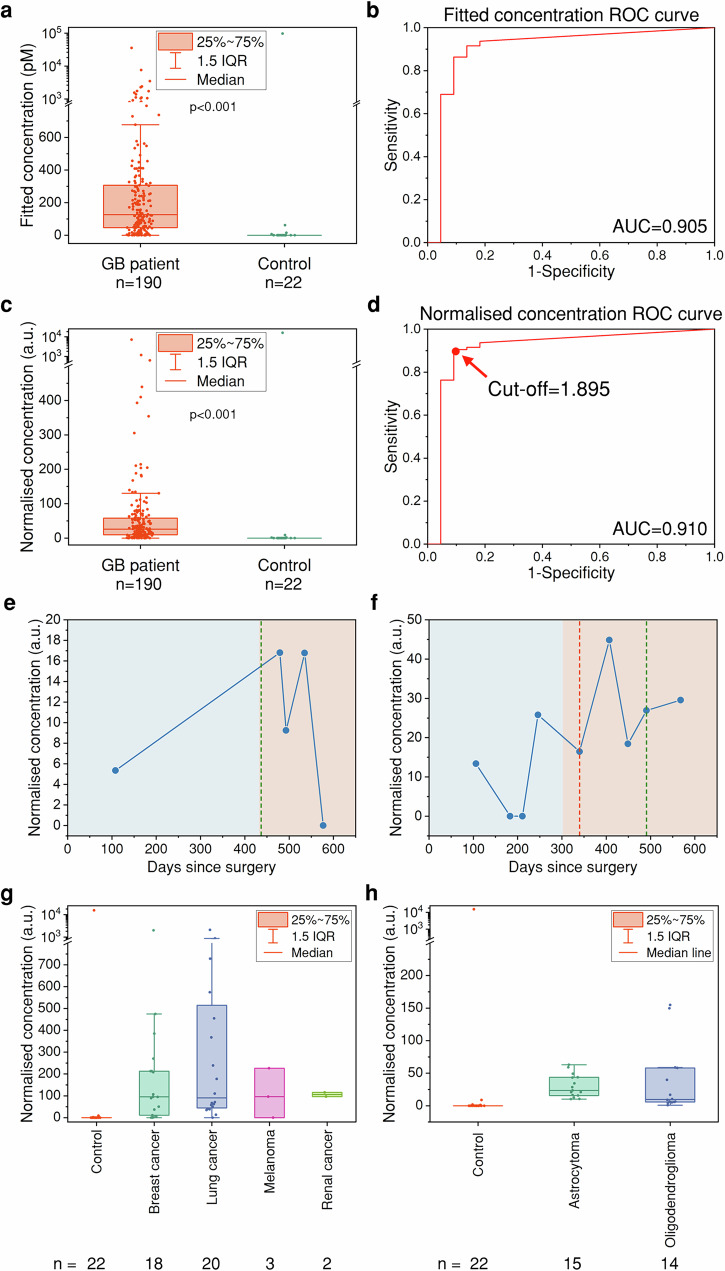
p53 aggregate concentrations across primary and metastatic brain cancers. **a** Fitted p53 aggregate concentrations of 190 GB patients vs 22 controls. **b** ROC curve plotted from fitted p53 aggregate concentrations. **c** Normalised p53 aggregate concentrations of 190 GB patients vs 22 controls. **d** ROC curve plotted from normalised p53 aggregate concentrations. **e**, **f** Representative results from longitudinal study of two post-operative GB patients, followed with serial MRI scans and plasma p53 levels. Initial rise in p53 aggregate levels prior to recurrence is followed by a reduction in p53 aggregate levels post-treatment. The blue chart area represents the period post-surgery during which follow-up MRI scans showed no evidence of recurrence, while the pink chart area represents the period following recurrence, including stable recurrence. Dotted lines depict the date of onset of chemotherapy (green) and further surgery (red). **g** p53 aggregate concentrations in the control vs metastatic brain cancers. **h** p53 aggregate concentrations in the control vs IDH-mutant primary cancer (astrocytoma and oligodendroglioma).

Using the optimised experimental conditions, we tested a total of 22 control and 190 GB patient plasma samples using the D-P antibody pair (see Table 1 for sample demographics). p53 aggregate concentrations were higher in the GB patients compared to controls (Control vs GB (Median (IQR)): 0 (0, 0) vs 126.100 (46.523, 308.072), Mann–Whitney *U*-test *p* value = 5.131 × 10^−10^, Fig. 4a). Notably, 18 of the 22 control samples (81.82%) had AEB values equal to or less than the blank AEB value on the same plate, and their concentrations were thus set to 0 pM. On the contrary, only 12 out of the 190 GB patients (6.32%) had undetectable p53 aggregate concentrations (0 pM). The ROC curve had an AUC of 0.905, demonstrating significant diagnostic accuracy (Fig. 4b). To ensure that the higher p53 aggregate concentrations detected in patient samples were not simply a result of elevated total protein concentrations, we further normalised the fitted p53 aggregate concentration to the total protein concentrations (as measured from A_280_, Supplementary Fig. S3) (Fig. 4c). The difference was still significant after normalisation (Control vs GB (Median (IQR)): 0 (0, 0) vs 26.003 (9.388, 58.321), Mann–Whitney *U*-test *p* value = 3.056 × 10^−10^), while the corresponding ROC curve showed a slightly higher AUC of 0.910 (Fig. 4d). The optimal threshold was determined by the Youden index to be 1.895 (a.u.)^38^, with which the diagnostic accuracy, sensitivity, and specificity were 90.57%, 90.53%, and 90.91%, respectively (Supplementary Fig. S4). *TP53* mutation status was available for 87 patients across this cohort. We observed no difference in p53 aggregate levels in plasma between those tumours that were mutant for *TP53* (*n* = 32) or wild type (*n* = 55) (Mutant vs WT (Median (IQR)): 31.77 (17.670, 54.535) vs 27.528 (11.206, 53.666), Mann–Whitney *U*-test *p* value = 0.318) (Supplementary Fig. S5). Only those patients with 2-hits were considered variant (1 patient was hemizygous for *TP53* mutation). These findings were consistent with transcriptome data where no significant difference was observed between the *TP53* transcript levels in GB patients’ tumour tissue with WT or mutant *TP53*; however, the *TP53* transcript levels were significantly higher in the tumour tissue of GB patients than in the normal tissue (Supplementary Fig. S6). We also examined normalised p53 aggregate concentrations in homogenised tumour tissue and observed no difference between patients with mutant *TP53* (*n* = 10) and WT *TP53* (*n* = 10) (Mutant vs WT (Median (IQR)): 224.596 (44.317, 1244.809) vs 126.025 (46.021, 425.174), Mann–Whitney *U*-test *p* value = 0.473) (Supplementary Fig. S7a). There was no significant correlation between the p53 aggregate concentrations in the plasma and brain samples of the same patient (Supplementary Fig. S7b).

**Table 1:** Sample demographics

	Controls (*n* = 22)	GB patients (*n* = 190)
Age (years, %)		
21–30	0 (0)	3 (1.6)
31–40	2 (9.1)	8 (4.2)
41–50	4 (18.2)	20 (10.5)
51–60	5 (22.7)	64 (33.7)
61–70	4 (18.2)	60 (31.6)
71–80	5 (22.7)	32 (16.8)
81–90	2 (9.1)	3 (1.6)
91–100	0 (0)	0 (0)
Sex		
Women (%)	9 (40.9)	69 (36.3)

Next, we identified 25 patients with GB for whom plasma samples were available at 2 or more post operative timepoints for inclusion in a retrospective longitudinal review. All patients underwent concurrent MRI enabling us to map the p53 aggregate measurement to the extent of radiological disease. p53 was detectable in all patient samples and radiological findings (stable disease vs tumour recurrence) were then mapped to subsequent treatment (surgery, radiotherapy and/or chemotherapy). Several patients had p53 aggregate concentration trajectories that showed consistency with their treatment record (Fig. 4e, f). Whilst these were broadly consistent with treatment response, variability exists and this may reflect the chemotherapeutic regimen used or the limited number of samples obtained and the opportunistic method by which the samples were collected.

### Detection of elevated p53 aggregate levels in the plasma of patients with IDH-mutant primary and metastatic brain cancers

Given the broad tumour-suppressing function of p53, we asked whether p53 aggregate levels are elevated in the plasma of other brain cancer patients, including IDH-mutant primary and metastatic brain cancers. Patients with metastatic lung cancer and breast cancer, all of whom had isolated brain metastatic lesions and stable disease elsewhere, showed comparable or higher plasma p53 aggregate concentrations than the GB samples (GB vs breast cancer vs lung cancer (Median (IQR)): 26.003 (9.388, 58.321) vs 95.160 (10.807, 213.070) vs 90.468 (44.214, 514.595)) (Fig. 4g), whereas patients with the lower grade IDH-mutant brain tumours, astrocytoma and oligodendroglioma, exhibited lower, but still significant, concentrations of p53 aggregates compared with GB (GB vs astrocytoma vs oligodendroglioma (Median (IQR)): 26.003 (9.388, 58.321) vs 23.568 (15.542, 43.751) vs 9.658 (5.780, 57.970)) (Fig. 4h). The plasma samples from patients with other brain metastases showed a variety of p53 aggregate concentrations; however, the sample sizes were too small to be considered statistically significant. These data suggest that elevated plasma p53 aggregate concentrations are not a specific indicator for GB and may be indicative of the presence of other cancers within the brain and possibly elsewhere in the body.

## Discussion

We have developed and optimised a SiMoA assay for quantifying p53 aggregates in human plasma samples. Using the DO-1 and PAb240 antibodies for capture and detection, the assay demonstrated a diagnostic accuracy of 90.57% in distinguishing between GB patients (*n* = 190) and controls (*n* = 22). The presence of p53 aggregates over 200 nm across was validated with super-resolution imaging using the same antibody pair. While we observed some consistency in p53 aggregate concentration and recurrence and treatment response in patients at the post-operative monitoring stage, this trend was not universal among all patients, and the opportunistic method for sample collection meant that only qualitative assessment could be performed. In addition, we observed increased p53 aggregate concentrations in cancer patients with brain metastases, suggesting that they may have diagnostic and prognostic applications beyond GB.

We observed no correlation between the presence/absence of *TP53* mutation and plasma concentration of p53 aggregates; there was also no significant difference between the p53 aggregate concentrations in the brain homogenate samples of GB patients with WT and mutant *TP53*. However, elevated p53 transcript and aggregates were observed in GB patients but not controls. These results are consistent with several studies that demonstrated the presence of WT p53 aggregates in cancer and we hypothesise that levels of p53 aggregation may reflect this^39^. WT p53, as a transcription factor, is responsible for the response to DNA damage^1^, hypoxia^40^, and oxidative stress^41^, all of which are found in brain cancer^42–44^. The concentration of WT p53 given the conditions associated with rapid tumour cell growth have been calculated to be above that required for aggregate seeding (18–180 nM) where the capacity for WT p53 to form aggregates at physiological concentrations (50–100 nM) has also been demonstrated albeit with lower efficiency than mutant *TP53*^34^. Increased accumulation of mutant and WT p53 has also been demonstrated in stressed cells^45^. We were able to demonstrate that irrespective of *TP53* mutational status, the expression of p53 is increased across a cohort of GB patient samples and that this is reflected by an increase in p53 aggregates identified from tumour homogenates.

The D-P antibody pair is the basis of the SiMoA assay. The DO-1 antibody-conjugated beads are thought to capture and enrich all p53 species, whereas the PAb240 antibody detects p53 aggregates that expose the unfolded DNA-binding domain. In contrast, while the D-D antibody pair is thought to detect multimeric p53, it did not show a clear separation between controls and GB patients, presumably due to the presence of p53 dimers and/or tetramers in the plasma of healthy individuals. However, while the presence of p53 aggregates in GB patient plasma has been validated by super-resolution imaging using the D-P antibody pair, it remains unclear whether the elevated SiMoA signals result purely from p53 aggregates, since the PAb240 antibody may also bind to unfolded p53 monomers. Further investigation is required to fully validate the exact species detected by SiMoA, as well as to characterise the size distribution of the detected p53 aggregates to shed light on their mechanism of production and biological roles. The p53 aggregate concentration trajectories showed broad correlation with the patients’ clinical profiles but these were sometimes difficult to interpret given the opportunistic method by which the samples were collected and the resultant gaps in longitudinal sampling. Also, we did not see a correlation between p53 aggregate concentration and survival within these patients, but this is perhaps unsurprising given the few patients for which we had multiple timepoints and the multifactorial nature of prognostication in glioma. A comprehensive correlation analysis between the patients’ p53 aggregate concentrations and clinical profiles is therefore needed to confirm the clinical implications of p53 aggregates, ideally for both pre-operative and follow-up patients; however, the multiplicity of prognostic factors in patients with brain cancer, such as the size and location of the tumour and the functional status of the patient, mandate a multivariant analysis with a larger numbers of patients included.

Our study highlights the potential utility of plasma p53 aggregate concentrations for early detection, diagnosis and monitoring of patients with brain cancer. Earlier detection of both primary and recurrent disease in GB and IDH mutant glioma would enable rapid institution of treatment and thereby maximise the potential for patient benefit. There is preliminary evidence that earlier detection of GB, in its low-grade state, may improve survival^46^. Given its high diagnostic accuracy, the p53 aggregate assay in the present study may serve as an early screening tool or assistive diagnostic method for GB; however, additional data are required to confirm the earliest time point possible for p53 aggregate to indicate GB development, and the optimal clinical threshold with balanced sensitivity and specificity. Monitoring GB recurrence may also bring important clinical benefit for GB patients, as GB ultimately recurs in all cases^9^. Importantly, detection of GB recurrence may reflect the effectiveness of ongoing therapeutic strategy; for example, discovering chemoresistance early may enable timely alternation of the treatment plan. Still, this demands in-depth investigation by correlating p53 aggregate concentrations with follow-up patients’ treatment records, such as chemotherapy, radiotherapy, and surgery, and other clinical characteristics (e.g., genetic information) in a larger cohort. Similarly, early detection of brain metastasis enables the rapid institution of a broad range of therapies that are not available when lesions are large and symptomatic. While we have discovered elevated p53 aggregate concentrations in several primary cancers with brain metastasis within a relatively small cohort, comprehensive correlation analyses within larger cohorts are needed to understand whether p53 aggregation is truly associated with brain metastasis.

Although MRI remains the primary method for identifying brain tumours, including at recurrence, alternate lower cost approaches, such as liquid biopsy^47,48^, that do not have the logistical challenges of MRI, and that avoid the high levels of patient anxiety whilst awaiting an MRI scan would be advantageous. Furthermore, the SiMoA assay is compatible with high-throughput testing and has a relatively low cost (~$20 per assay) compared with techniques that detect other cancer biomarkers, such as circulating tumour DNA (ctDNA) sequencing. Therefore, the p53 aggregate assay holds the potential to be widely adopted in clinics and healthcare systems for large-scale screening of cancer, and may help clinicians in primary care refer potential cancer patients for further medical examinations.

There are several limitations with this study. Firstly, there are a few outliers with exceptionally high AEB values in both the control and GB patient groups, which cannot be explained by their clinical profiles. The elevated AEB values may result from contamination during sample handling, or the presence of human anti-mouse antibodies in these samples^49^. These antibodies in the samples may bind to the capture and detector antibodies, both of which are mouse-origin, and lead to abnormally high AEB values. However, more investigations are needed to examine these hypotheses. Secondly, the specific p53 species detected by the SiMoA assay have not been fully resolved. While its diagnostic or prognostic value may be unaffected, understanding the population of unfolded p53 or p53 aggregates in plasma, as well as their structure and source, may provide fundamental insights into the development of GB and other cancers. Thirdly, the fact that p53 aggregates can be detected in IDH mutant primary and metastatic brain tumours indicates that the presence of p53 aggregate is not an exclusive biomarker for GB diagnosis. Hence, additional blood biomarkers need to be developed and used in combination with p53 aggregates to enhance the diagnostic specificity. Glioma has been shown previously to be a particularly challenging cancer to detect through liquid biopsy and as such, these data may indicate broader applicability of this approach across cancers in which liquid biopsy is more straightforward. In addition, given the broad tumour-suppressing functions of p53, p53 aggregates may be expected to be present in the blood plasma of patients with various cancers other than primary brain cancer, especially those with *TP53* mutations. Thus, the prevalence of *TP53* mutation across cancers and the broad applicability of p53 aggregate detection may enable pan-cancer diagnostic tests to be developed.

## Supplementary information

Supplementary information

Description of Additional Supplementary files

Supplementary data 1

Supplementary data 2

Supplementary data 3

Reporting Summary

## Data Availability

The SiMoA data are included in Supplementary Data 1. The Source data presented in all figures are in Supplementary Data 2. All fluorescence imaging data are available upon request. The *TP53* sequencing data in this paper forms part of a larger Minderoo Precision Brain Tumour Program. The raw *TP53* data as well as any data transformations have been shared as a csv file in Supplementary Data 3.
