# Supplementary material for: Detection of p53 aggregates in plasma of glioma patients
Source: Commun Med (Lond). 2025 May 23;5:195. doi: 10.1038/s43856-025-00918-3 (PMC12102397; doi:10.1038/s43856-025-00918-3)
Supplement: Supplementary file 1 — Supplementary information [file 43856_2025_918_MOESM1_ESM.pdf]

Supplementary Information

Detection of p53 aggregates in plasma of glioma patients

Yunzhao Wu[1,2], Jeff Y.L. Lam[1,2], Matthaios Pitoulias[3], Dorothea Böken[1,2], Ziwei Zhang[1,2], Renuka Chintapalli[3], Emre Fertan[1,2], Zengjie Xia[1,2], John S.H. Danial[1,2], Gemma Tsang-Pells[3], Emily Fysh[3], Linda Julian[1,4], Kevin M. Brindle[4], Richard Mair[3,4]*, David Klenerman[1,2]*.

1 Yusuf Hamied Department of Chemistry, University of Cambridge, Cambridge CB2 1EW, United Kingdom

2 UK Dementia Research Institute, University of Cambridge, Cambridge CB2 0AH, United Kingdom

3 Department of Clinical Neurosciences, University of Cambridge, Cambridge CB2 0QQ, United Kingdom

4 Cancer Research UK Cambridge Institute, University of Cambridge, Cambridge CB2 0RE, United Kingdom

*Co-corresponding authors

Supplementary Table S1. Diagnoses of the control group.

Disease	Number of controls
Cerebral artery aneurysm, unruptured	3
Cervical myelopathy	4
Hydrocephalus	3
Lumbar radiculopathy	10
Parkinson’s disease	1
Trigeminal neuralgia	1

Supplementary Table S2. Lower detection limit and AEB range for different detector-SBG combinations.

Entry	PAb240 detector concentration (µg/mL)	SBG concentration (pM)	Lower limit of detection (pM)	AEB at highest calibrator concentration	Background AEB
**1**	0.3	50	10.17	0.63	0.0060
**2**	0.3	150	11.03	0.84	0.0098
**3**	0.3	300	10.35	1.10	0.0136
**4**	0.5	50	9.78	0.75	0.0068
**5**	0.5	150	11.85	0.89	0.0115
**6**	0.5	300	15.41	1.14	0.0179
**7**	1.0	50	10.35	0.88	0.0100
**8**	1.0	150	7.98	1.13	0.0124
**9**	1.0	300	18.84	1.12	0.0269

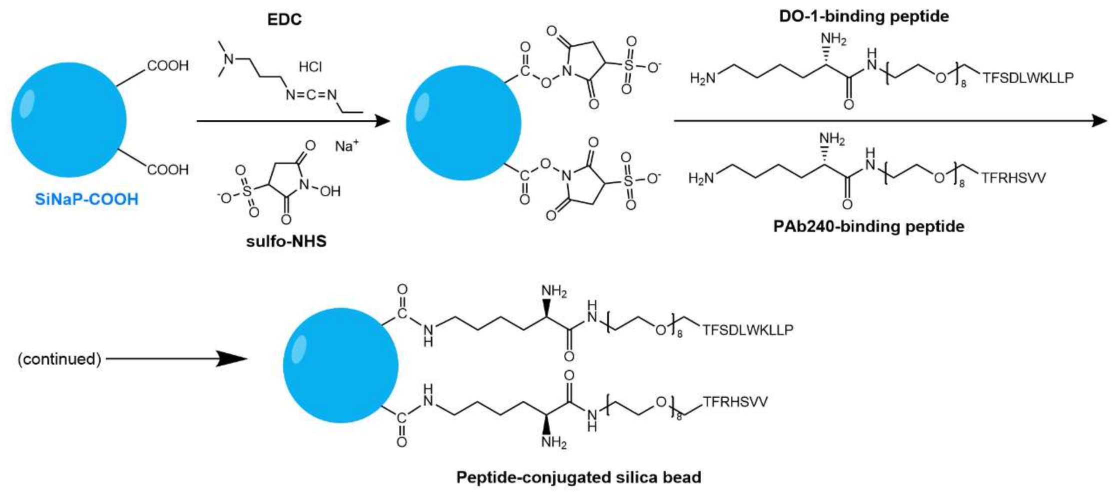

Supplementary Figure S1. Synthesis of the calibrator beads.

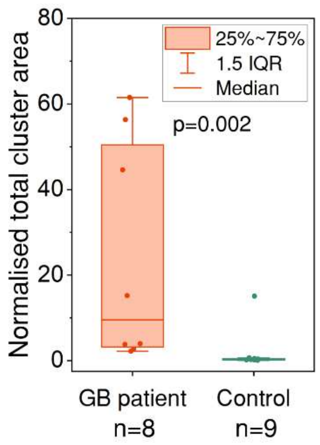

Supplementary Figure S2. Normalised total cluster area in the super-resolution images of GB patients and controls. The data were normalised to the corresponding values of the blank wells on the same coverslip.

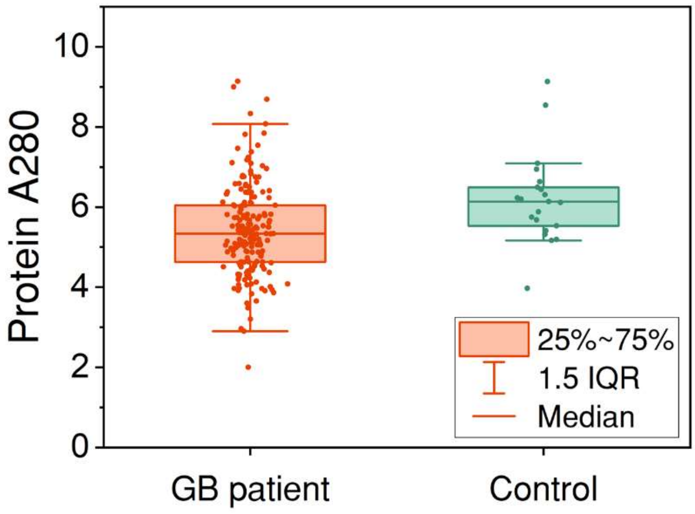

Supplementary Figure S3. Protein A280 of the GBM patient and control plasma samples.

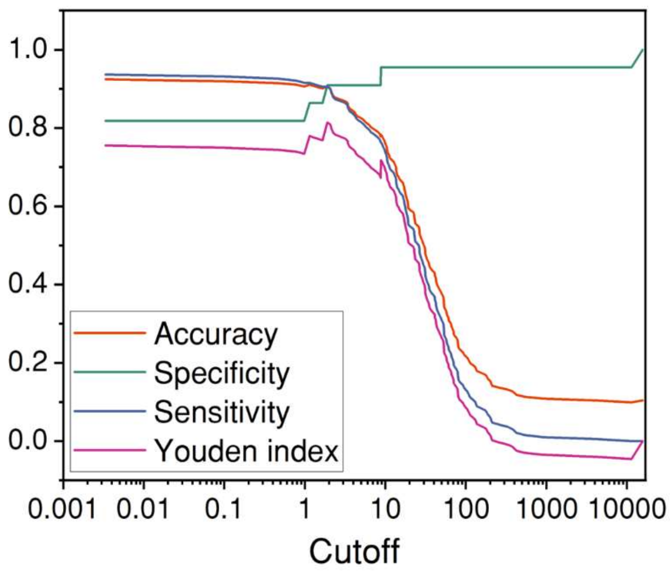

Supplementary Figure S4. Diagnostic accuracy, specificity, sensitivity, and Youden indices at different cut-offs.

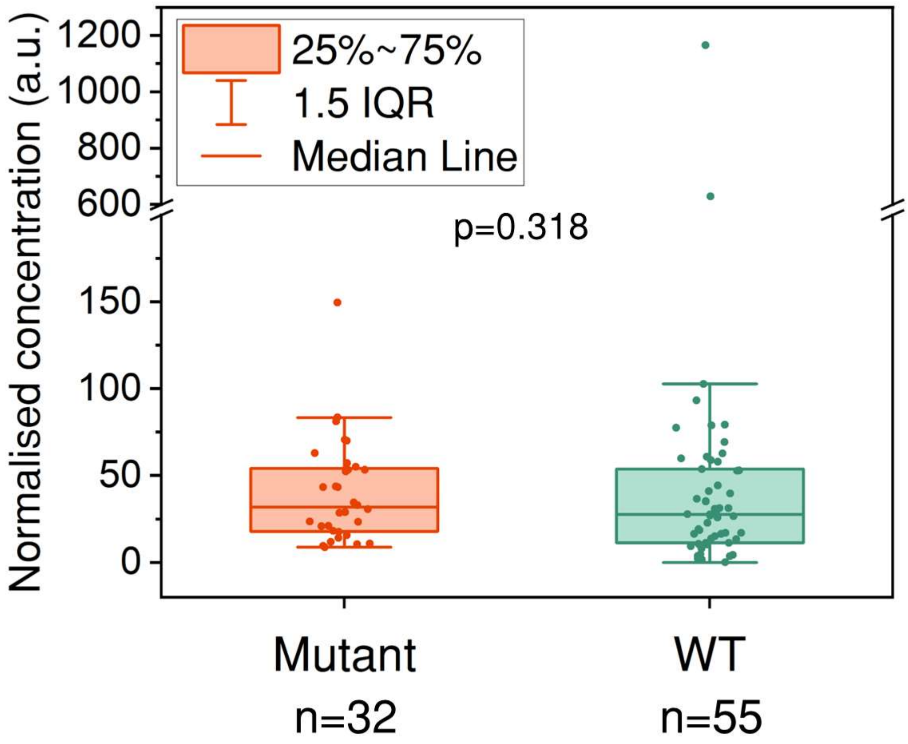

Supplementary Figure S5. Comparison between normalised p53 aggregate concentrations in the plasma samples of GB patients with mutant and WT *TP53*. The p53 aggregate concentrations were normalised by the total protein concentration in the plasma samples (as in protein A280).

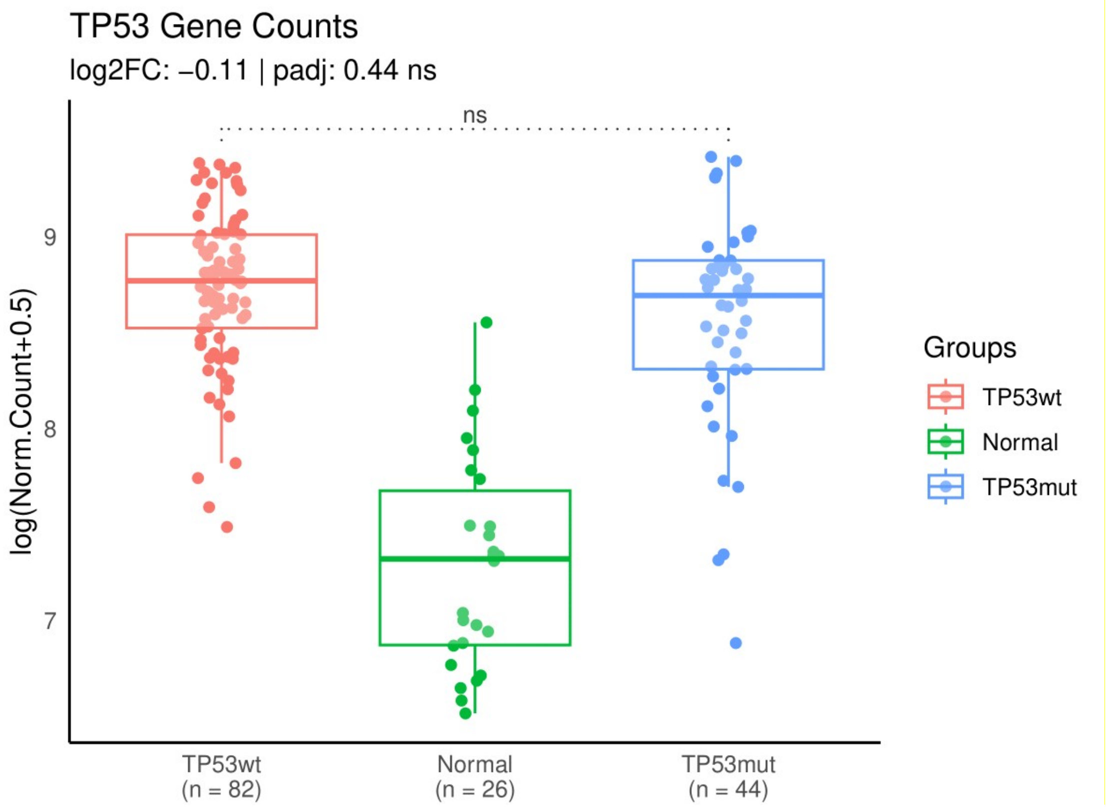

Supplementary Figure S6. Levels of p53 transcripts in GB patients' tumour and normal brain tissue samples. There was no difference between tumour samples with WT and mutant *TP53*. The p53 transcript levels in the normal brain tissue were significantly lower than those in the tumour tissue.

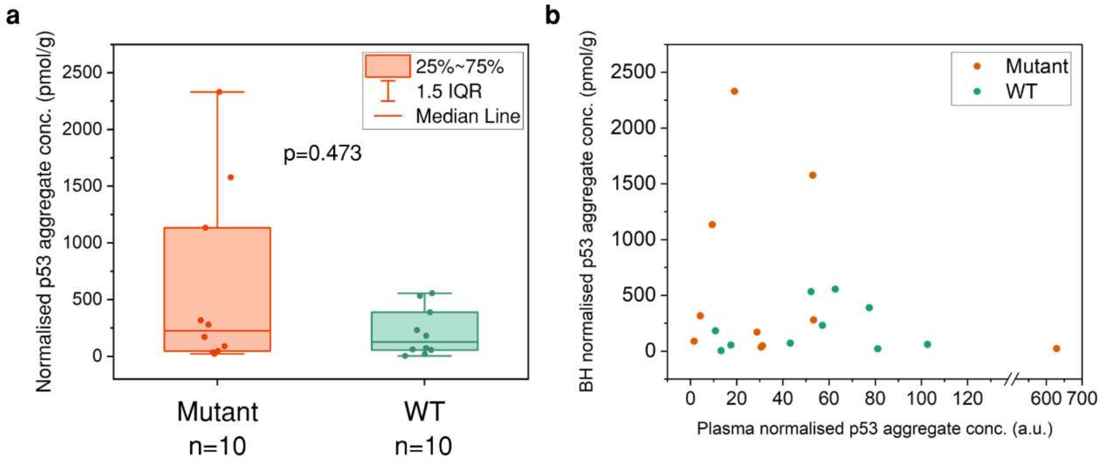

Supplementary Figure S7. P53 aggregate concentrations in brain homogenate samples. (a) Comparison between normalised p53 aggregate concentrations in the brain homogenate samples of GB patients with mutant and WT *TP53*. The p53 aggregate concentrations (pM) were normalised by total protein concentration in the brain homogenate samples (as measured by BCA method, in mg/mL). (b) No significant correlation was found between the p53 aggregate concentrations in the brain homogenate and plasma of the same patient (Pearson correlation p=0.477, Spearman correlation p=0.622). Each point represents one patient. BH, brain homogenate.
