# Supplementary material for: Detection of p53 aggregates in plasma of glioma patients
Source: Commun Med (Lond). 2025 May 23;5:195. doi: 10.1038/s43856-025-00918-3 (PMC12102397; doi:10.1038/s43856-025-00918-3)
Supplement: Supplementary file 2 — Description of Additional Supplementary files [file 43856_2025_918_MOESM2_ESM.pdf]

Description of Additional Supplementary files

File name: Supplementary Data 1

Supplementary data 1 are the source data for SiMoA data.

File name: Supplementary Data 2

Supplementary Data 2 contains the source data presented in all figures.

File name: Supplementary Data 3

Supplementary data 3 contains the raw TP53 data as well as any data transformations have been shared as a csv file.
