# Supplementary material for: Detection of p53 aggregates in plasma of glioma patients
Source: Commun Med (Lond). 2025 May 23;5:195. doi: 10.1038/s43856-025-00918-3 (PMC12102397; doi:10.1038/s43856-025-00918-3)
Supplement: Supplementary file 6 — Reporting Summary [file 43856_2025_918_MOESM6_ESM.pdf]

nature portfolio

Corresponding author(s): David Klenerman

Last updated by author(s): May 1, 2025

# Reporting Summary

Nature Portfolio wishes to improve the reproducibility of the work that we publish. This form provides structure for consistency and transparency in reporting. For further information on Nature Portfolio policies, see our Editorial Policies and the Editorial Policy Checklist.

## Statistics

For all statistical analyses, confirm that the following items are present in the figure legend, table legend, main text, or Methods section.

| n/a | Confirmed | |
|---|---|---|
| ☐ | ☒ | The exact sample size (*n*) for each experimental group/condition, given as a discrete number and unit of measurement |
| ☐ | ☒ | A statement on whether measurements were taken from distinct samples or whether the same sample was measured repeatedly |
| ☐ | ☒ | The statistical test(s) used AND whether they are one- or two-sided<br>*Only common tests should be described solely by name; describe more complex techniques in the Methods section.* |
| ☒ | ☐ | A description of all covariates tested |
| ☒ | ☐ | A description of any assumptions or corrections, such as tests of normality and adjustment for multiple comparisons |
| ☐ | ☒ | A full description of the statistical parameters including central tendency (e.g. means) or other basic estimates (e.g. regression coefficient) AND variation (e.g. standard deviation) or associated estimates of uncertainty (e.g. confidence intervals) |
| ☐ | ☒ | For null hypothesis testing, the test statistic (e.g. *F*, *t*, *r*) with confidence intervals, effect sizes, degrees of freedom and *P* value noted<br>*Give P values as exact values whenever suitable.* |
| ☒ | ☐ | For Bayesian analysis, information on the choice of priors and Markov chain Monte Carlo settings |
| ☒ | ☐ | For hierarchical and complex designs, identification of the appropriate level for tests and full reporting of outcomes |
| ☒ | ☐ | Estimates of effect sizes (e.g. Cohen's *d*, Pearson's *r*), indicating how they were calculated |

*Our web collection on statistics for biologists contains articles on many of the points above.*

## Software and code

Policy information about availability of computer code

| | |
|---|---|
| Data collection | The SiMoA data were collected by the SiMoA SR-X machine manufactured by Quanterix. The fluorescence images were collected with a home-built microscope using the MicroManager software. |
| Data analysis | The super-resolution images were analysed using the ThunderSTORM plugin in ImageJ/FIJI. |

For manuscripts utilizing custom algorithms or software that are central to the research but not yet described in published literature, software must be made available to editors and reviewers. We strongly encourage code deposition in a community repository (e.g. GitHub). See the Nature Portfolio guidelines for submitting code & software for further information.

## Data

Policy information about availability of data

All manuscripts must include a data availability statement. This statement should provide the following information, where applicable:

- Accession codes, unique identifiers, or web links for publicly available datasets
- A description of any restrictions on data availability
- For clinical datasets or third party data, please ensure that the statement adheres to our policy

The SiMoA data are included in Supplementary Data 1. The source data presented in all figures are in Supplementary Data 2. All fluorescence imaging data are available upon request. The TP53 sequencing data in this paper forms part of larger Minderoo Precision Brain Tumour Program. The raw TP53 data as well as any data transformations have been shared as a csv file in Supplementary Data 3.

## Human research participants

Policy information about studies involving human research participants and Sex and Gender in Research.

| | |
|---|---|
| Reporting on sex and gender | 'Sex' was used to discribe the biological sex of all participants. A total of 190 glioblastoma patients (of which 69 were women) and 22 controls (of which 9 were women) were included in this study. Sex was not considered when recruiting the patients. |
| Population characteristics | A total of 190 glioblastoma patients (of which 69 were women) and 22 controls (of which 9 were women) were included in this study. The age distribution is shown in Table 1. |
| Recruitment | Patients were recruited at Addenbrooke's Hospital, Cambridge, UK as part of the Integrated Clinically-Augmented Repository for Universal Sampling (ICARUS) tissue collection (REC 18/EE/0172). |
| Ethics oversight | University of Cambridge |

Note that full information on the approval of the study protocol must also be provided in the manuscript.

# Field-specific reporting

Please select the one below that is the best fit for your research. If you are not sure, read the appropriate sections before making your selection.

☒ Life sciences ☐ Behavioural & social sciences ☐ Ecological, evolutionary & environmental sciences

For a reference copy of the document with all sections, see nature.com/documents/nr-reporting-summary-flat.pdf

# Life sciences study design

All studies must disclose on these points even when the disclosure is negative.

| | |
|---|---|
| Sample size | The minimum sample size was determined to be 16 patients + 16 controls using preliminary data (Cohen's d, power = 0.95, alpha = 0.05). The actual sample size is 190 patients + 22 controls as this is the number of available samples. |
| Data exclusions | No data were excluded. |
| Replication | In calibrator development, three repeats of three on-plate replicates were performed. In SiMoA experiments, all patient samples were tested in two duplicate wells on the same plate. |
| Randomization | No randomization was performed in this study. Patients with suspected GB on pre-operative contrast-enhanced MRI were chosen for participation in the study. Sampling was performed during the initial surgery for a new diagnosis of glioma. Plasma from control patients undergoing non-cancer surgery was collected intra-operatively (matching conditions for the GB cohort). |
| Blinding | The investigator was not blinded during the research because the samples needed to be revealed for assay development. |

# Reporting for specific materials, systems and methods

We require information from authors about some types of materials, experimental systems and methods used in many studies. Here, indicate whether each material, system or method listed is relevant to your study. If you are not sure if a list item applies to your research, read the appropriate section before selecting a response.

## Materials & experimental systems

| n/a | Involved in the study | |
|---|---|---|
| ☐ | ☒ | Antibodies |
| ☒ | ☐ | Eukaryotic cell lines |
| ☒ | ☐ | Palaeontology and archaeology |
| ☒ | ☐ | Animals and other organisms |
| ☒ | ☐ | Clinical data |
| ☒ | ☐ | Dual use research of concern |

## Methods

| n/a | Involved in the study | |
|---|---|---|
| ☒ | ☐ | ChIP-seq |
| ☒ | ☐ | Flow cytometry |
| ☒ | ☐ | MRI-based neuroimaging |

## Antibodies

| | |
|---|---|
| Antibodies used | DO-1 antibody, Abcam, Cat. No. Ab1101; PAb240-biotin antibody, Novus Biologicals, Cat. No. NB200-103B |

| | |
|---|---|
| Validation | DO-1 was knock-out validated (claimed by Abcam). Validated applications of PAb240 included Western blotting, ELISA, flow cytometry, immunohistochemistry, and so on (claimed by Novus Biologicals). |
